# New zoonotic cases of *Onchocerca dewittei japonica* (Nematoda: Onchocercidae) in Honshu, Japan

**DOI:** 10.1186/s13071-015-0655-2

**Published:** 2015-01-27

**Authors:** Shigehiko Uni, Masako Fukuda, Yasushi Otsuka, Nobuo Hiramatsu, Kenichi Yokobayashi, Hiroshi Takahashi, Susumu Murata, Kenji Kusatake, Eishin Morita, Haruhiko Maruyama, Hideo Hasegawa, Kuninori Shiwaku, Rosli Ramli, Mohd Sofian Azirun, Hiroyuki Takaoka

**Affiliations:** Institute of Biological Sciences, Faculty of Science, University of Malaya, 50603 Kuala Lumpur, Malaysia; Department of Parasitology, Graduate School of Medicine, Osaka City University, Osaka, 545-8585 Japan; Research Promotion Institute, Oita University, Oita, 879-5593 Japan; Research Center for the Pacific Islands, Kagoshima University, Kagoshima, 890-8580 Japan; Hiramatsu Orthopedic Clinic, Hiroshima, 730-0016 Japan; Hiroshima University Hospital, Hiroshima, 734-8551 Japan; Department of Dermatology, Faculty of Medicine, Shimane University, Shimane, 693-8501 Japan; Department of Infectious Diseases, Division of Parasitology, Faculty of Medicine, University of Miyazaki, Miyazaki, 889-1692 Japan; Department of Biology, Faculty of Medicine, Oita University, Oita, 879-5593 Japan; Department of Environmental and Preventive Medicine, Faculty of Medicine, Shimane University, Shimane, 693-8501 Japan

**Keywords:** Filarioid, Global warming, Japanese wild boar, *Onchocerca dewittei japonica*, Vector-borne disease, Zoonosis

## Abstract

**Background:**

Zoonotic infections with *Onchocerca* species are uncommon, and to date only 25 clinical cases have been reported worldwide. In Japan, five previous zoonotic infections were concentrated in Oita, Kyushu (the southern island), with one previous case in Hiroshima in the western part of Honshu (the main island). The causative agent in Japan was identified as *Onchocerca dewittei japonica* Uni, Bain & Takaoka, 2001 from Japanese wild boars (*Sus scrofa leucomystax* Temminck, 1842). Here we report two infections caused by a female and male *O. dewittei japonica*, respectively, among residents of Hiroshima and Shimane Prefectures in the western part of Honshu.

**Methods:**

In both cases, nodules were surgically removed. The parasites in nodules were identified on the basis of their histopathological characteristics. Identification was confirmed by sequencing the mitochondrial cytochrome *c* oxidase subunit 1 (*cox*1) gene from worms in the tissues used in the histological preparations.

**Results:**

Case 1 was a 61-year-old woman from Hiroshima Prefecture who complained of a painful subcutaneous nodule on the back of her right hand. The causative agent was identified as a female *O. dewittei japonica* owing to transverse ridges on the cuticle and molecular analysis. Case 2 was a 78-year-old woman from Shimane Prefecture who had a painful nodule in the left temporal region. Histopathological characteristics and *cox*1 sequencing of the worm indicated that the causative agent was a male *O. dewittei japonica*.

**Conclusions:**

For Cases 1 and 2, we diagnosed the causative agents as a female and male *O. dewittei japonica*, respectively. These findings indicate the spread of a zoonosis caused by *O. dewittei japonica* in the western part of Honshu, where wild boars have recently extended their habitats because of decreased annual snowfall, unused rice fields and a decline in the number of hunters in Japan. The *O. dewittei japonica* infection rate among wild boars was reported as 78% in Shimane Prefecture, in the western part of Honshu. Therefore, in the near future, zoonotic onchocercosis is likely to occur in Honshu as well as Kyushu, where wild boars, blackfly vectors and humans share the same habitat.

## Background

Zoonotic filariosis is a human infection caused by animal filarioids, which are transmitted by blood-sucking vectors. The reported incidence of vector-borne parasitic zoonoses has recently increased throughout the world. The alterations in climate (particularly global warming), deforestation, urbanisation and human demographics have affected the transmission of parasites among vectors, host animals and humans. These factors have led to the occurrence of vector-borne parasitic zoonoses in areas where such infections have not been previously reported in humans [1-4].

Twenty-five clinical cases caused by *Onchocerca* spp. transmitted from animals have been reported worldwide: eight in North America, six in Japan, five in Europe, three in Turkey, one in Kuwait, one in Tunisia and one in Iran. Among these, five ocular infections and one cervical spinal mass caused by *Onchocerca lupi* Rodonaja, 1967 were recently reported in Turkey, Tunisia, USA and Iran [5-14]. Five suspected or identified causative agents were *O. gutturosa* Neumann, 1910 from cattle, *O. cervicalis* Railliet & Henry, 1910 from horses, *O. dewittei japonica* from Japanese wild boars, *O. jakutensis* (Gubanov, 1964) from European deer and *O. lupi* from carnivores (e.g. dogs) [7,15-18].

In Japan, seven *Onchocerca* species (*O. gutturosa*, *O. lienalis* Stiles, 1892, *O. cervicalis, O. suzukii* Yagi, Bain & Shoho, 1994, *O. dewittei japonica*, *O. skrjabini* Rukhlyadev, 1964 and *O. eberhardi* Uni & Bain, 2007) have been identified in domestic and wild animals [4,19-21]. Two unnamed *Onchocerca* species (*Onchocerca* sp. from wild boars and *Onchocerca* sp. Type A from blackflies) were recently differentiated from other congeneric species by molecular analyses [22-24]. Six clinical cases of zoonotic onchocercosis have been reported from Japan, where five infections were concentrated in Oita, Kyushu (the southern island), and another case occurred in Hiroshima in the western part of Honshu (the main island) [5]. The blackfly *Simulium bidentatum* (Shiraki, 1935), anthropophilic and zoophilic, was verified as a natural vector of *O. dewittei japonica* in Oita, Kyushu [22-24] and the blackfly vectors have been found in Honshu [4].

In the current study, we present two cases of *O. dewittei japonica* infections in the western part of Honshu that were identified on the basis of their histopathological and molecular characteristics. These findings indicate that zoonotic infections caused by *O. dewittei japonica* have occurred in the western part of Honshu, owing to the increase in numbers and the habitat expansion of wild boars in Honshu as well as Kyushu [25]. The nomenclature of parasitic diseases follows the guideline proposed by the Executive Committee of the World Association for the Advancement of Veterinary Parasitology [26].

## Methods

### Clinical history

Case 1 was a 61-year-old woman from the Higashihiroshima City, Hiroshima Prefecture, Japan. The patient was a housewife who lived in a rural area near mountains inhabited by wild boars. She often observed blackflies and had a dog as a pet. She had not travelled outside Japan during the previous 10 years. In November 2010, she developed a painful nodule on the back of her right hand. On 25 November, the nodule was surgically removed at a hospital in Hiroshima. After the surgery, she was examined for the parasitic infections at Hiroshima University Hospital until February 2011 and no signs were found.

Case 2 was a 78-year-old woman from Izumo City, Shimane Prefecture, Japan. The patient lived in a rural area near mountains inhabited by wild boars. She occasionally worked outside as a farmer. She reported that she had been bitten by blackflies and mosquitoes. She had never travelled outside Japan and had not visited Kyushu for 10 years. She had a cat as a pet. In January 2011, she developed a nodule in the left temporal region of her head. She reported that the nodule caused pain for 2 weeks and was increasing in size. On 1 April 2011, the nodule was surgically removed at Shimane Medical University Hospital. After this, she was treated with antibiotics and analgesics for 3 days. For 4 months after the surgery, her head, thoracic, abdominal and pelvic areas were continuously examined by computed tomography scan for any parasitic infections. The results showed that there were no lesions. Eosinophilia in the patient decreased from 10.2% after the surgery on 19 April 2011 to 4.2% (the normal value) on 29 June 2011. Laboratory examinations detected no immunological deficiencies.

### Histopathological and molecular analyses

**Figure 1:**
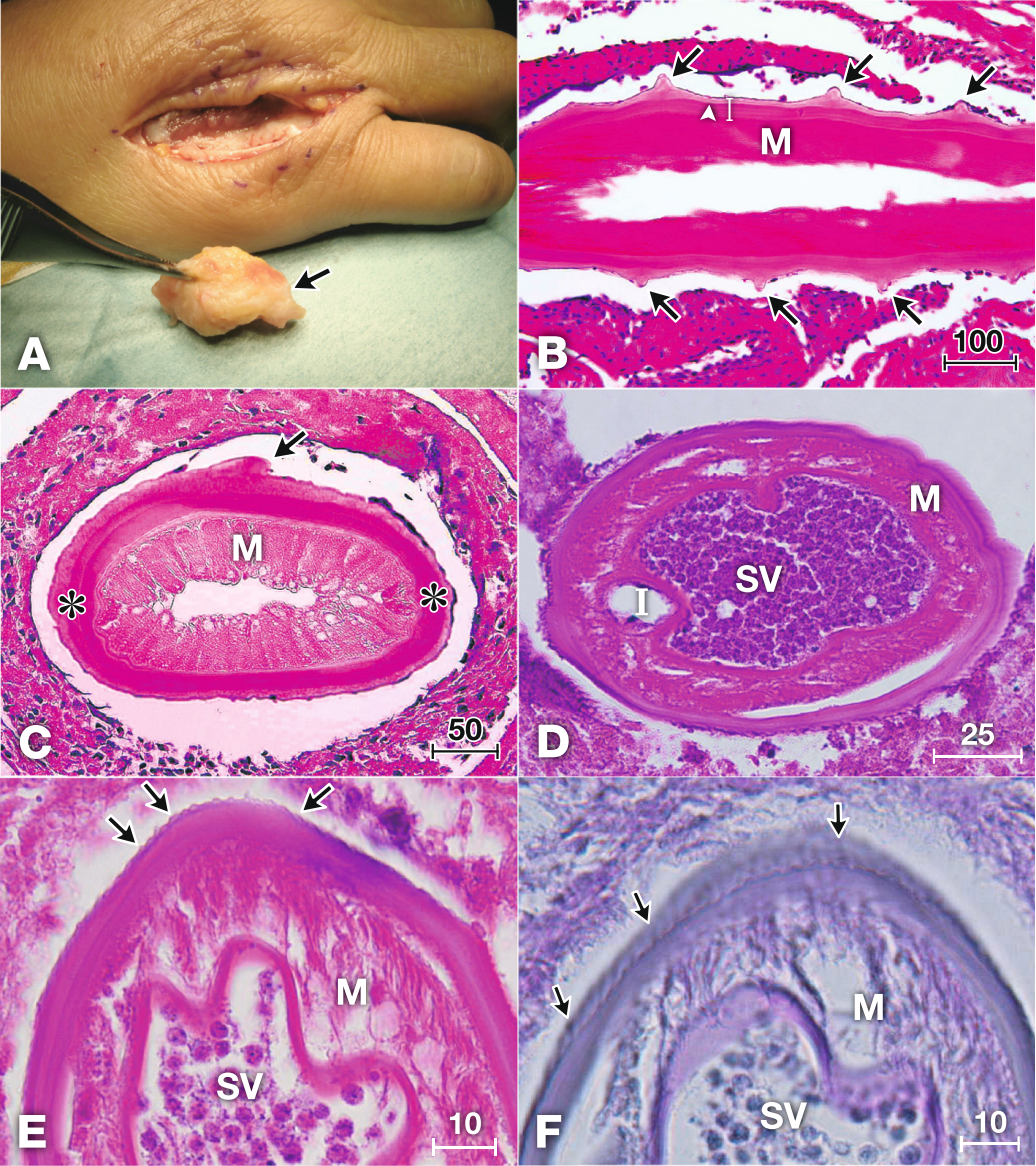
**Histopathological characteristics of** ***Onchocerca dewittei japonica*** **in nodules excised from Cases 1 in Hiroshima (A–C) and 2 in Shimane (D–F). A)** Mass (arrow, 1 × 3 cm) excised from the back of the right hand. **B)** Longitudinal section of the female *O. dewittei japonica*. Arrows, triangular transverse ridges; arrowhead, middle layer of the cuticle without the inner striae; white vertical line, cuticle; M, muscle layer. HE staining. **C)** Slightly oblique transverse section of the female worm showing the thick cuticle. Arrow, elevation of the cuticle, indicating the transverse ridge; asterisks, lateral chords; M, muscle layer. HE staining. **D)** Transverse section of the male *O. dewittei japonica*. I, intestine; M, muscle layer; SV, seminal vesicle with spermatozoids. HE staining. **E)** Enlarged transverse section. Arrows, small ridges on the cuticle; M, muscle layer; SV, seminal vesicle. HE staining. **F)** Slightly oblique transverse section. Arrows, small longitudinal ridges; M, muscle layer; SV, seminal vesicle. PAS staining. Unit of bars, μm.

For Case 1, the excised mass (1 × 3 cm, Figure 1A) was fixed in 10% buffered formalin for several hours and embedded in paraffin. Sections (3 μm thick) were stained with haematoxylin and eosin (HE). Microscopic examinations revealed five longitudinal sections, two oblique sections and five transverse sections of a worm. Five histological sections stained with HE were used for the molecular analysis. These sections were immersed in xylene for 2-3 days to remove the cover glass. The worm tissues (0.6 mm^2^) were scraped off with a sterile scalpel under a stereomicroscope for use in the molecular analysis. The tissues were used to determine the nucleotide sequence of the mitochondrial cytochrome *c* oxidase subunit 1 (*cox*1) gene. The tissues were incubated with 0.5 mL of DEXPAT (Takara Bio Inc., Otsu, Japan) for 10 min at 100°C and centrifuged for 10 min at 12,000 rpm at 4°C. The supernatants containing the extracted DNA were mixed and concentrated by ethanol precipitation. Approximately 30 ng of DNA was used as a template for PCR. PCR amplification was performed using the primers CO1fF-CO1fR (expected size: 239 bp) as described previously [6]. However, the primers failed to amplify *cox*1 from our specimens; therefore, we designed a new reverse primer called CO1f1R (5′- AAAATAATAACATAAACCTCAGGATG-3′) and used a new primer set CO1fF-CO1f1R for amplification. The position of the primer in the complete mitochondrial genome of *O. volvulus* [GenBank: AF015193] is 3013–3038. The thermal conditions were as follows: initial denaturation at 94°C for 2 min, followed by 40 cycles at 98°C for 10 s, 47°C for 30 s and 68°C for 30 s. The PCR product of the expected size (155 bp) was excised from the agarose gel, purified with a QIAEX II Gel Extraction Kit (Qiagen, Hilden, Germany) and cloned into the *Hinc*II site of the pUC118 plasmid vector with a Mighty Cloning Reagent Set < Blunt End > (Takara Bio Inc.). The inserted fragments from six colonies were sequenced using M13F (-20) and M13R primers, a BigDye Terminator v3.1 Cycle Sequencing Kit (Applied Biosystems, Foster City, CA, USA) and an Applied Biosystems 3130 Genetic Analyzer (Applied Biosystems).

The nucleotide sequence was aligned with the published sequences of 11 *Onchocerca* species using CLUSTAL W with the default settings in BioEdit ver. 7.0.5.3. [27,28]. Two unnamed species (*Onchocerca* sp. wild boar and *Onchocerca* sp. Type A) were included in the comparison. Two other filarial species (*Loxodontofilaria caprini* Uni & Bain, 2006 and *Cercopithifilaria longa* Uni, Bain & Takaoka, 2002) were used as outgroups. The Kimura two-parameter method [29] was used to estimate evolutionary distances from the alignments. The phylogenetic tree was constructed using the neighbour-joining method [30], and the bootstrap probabilities were estimated. The analyses were performed based on 110 sites of the *cox*1 using MEGA 5.05 [31].

For Case 2, the excised mass (1 × 1.5 cm) was fixed in 10% buffered formalin for 6 h and embedded in paraffin. To obtain morphological observations, four sections on one glass slide were stained with HE, and four sections on another glass slide underwent periodic acid–Schiff (PAS) staining. In the molecular analysis, 30 sections (5 μm thick) of the worm tissues were obtained from the paraffin block, and the unstained worm tissues (0.11 mm^2^ in 10 sections) were used. The scraped worm tissues were transferred into nine micro-centrifuge tubes. The DNA extraction procedure and PCR amplification using the primer set CO1fF-CO1f1R were performed as described above. Template DNA (0.8 μg) was amplified under the following thermal conditions: initial denaturation at 94°C for 2 min, followed by 45 cycles at 98°C for 10 s, 42°C for 30 s and 68°C for 30 s. The procedures used to sequence the PCR products and analyse the data were performed as described above. The sequences determined in Cases 1 and 2 were deposited in DDBJ/EMBL/GenBank.

## Results

In the longitudinal sections of Case 1, the worm exhibited transverse cuticular ridges, which are typical of female *Onchocerca* species (Table 1 and Figure 1B). Each ridge formed a triangular projection in the longitudinal sections, and the distance between two adjacent ridges was 150–250 μm (arrows in Figure 1B). There were no inner striae in the middle layer of the cuticle (arrowhead in Figure 1B). The cuticle was thick and consisted of four layers. The slightly oblique transverse sections were oval and measured 125–153 × 278–280 μm (Figure 1C). An elevated portion of the cuticle indicated the presence of a ridge (arrow in Figure 1C). The cuticle, hypodermis, lateral chords and polymyarian muscle layer were observed; however, the uterus and intestine were lost during the preparation of the sections. There was no inner cuticular projection on the lateral chord, but irregular waves were observed in the hypodermis. In terms of the host–worm interaction, the worm was surrounded by fibrous tissue, containing macrophages, neutrophils, eosinophils and lymphocytes. In conclusion, the morphological characteristics of the worm were identical or very similar to those of adult females of *O. dewittei japonica* collected from wild boars (Table 1).

**Table 1:** **Comparisons of the histological characteristics of a female and male of** ***Onchocerca dewittei japonica*** **, obtained from human cases in the current study, with congeneric species from Japan**

	***Onchocerca dewittei japonica*** **Uni, Bain & Takaoka, 2001**	***Onchocerca dewittei japonica***	***Onchocerca dewittei japonica***	***Onchocerca gutturosa*** **Neumann, 1910**	***Onchocerca lienalis*** **Stiles,1892**	***Onchocerca skrjabini*** **Rukhlyadev, 1964**	***Onchocerca eberhardi*** **Uni & Bain, 2007**	***Onchocerca cervicalis*** **Railliet & Henry, 1910**	***Onchocerca suzukii*** **Yagi, Bain & Shoho, 1994**
References	Case 1 in the current study	Case 2 in the current study	[20]	[32]; *[33]; **[34]	[32]; ***[17]	[19]; ****[5]	[21]; ****[5]	[36]; *****[33]	[19]; ******[5]
Host(s)	Human in Hiroshima	Human in Shimane	*Sus scrofa leucomystax* Temminck, 1842 (Japanese wild boar)	Cattle	Cattle	*Cervus nippon* Temminck, 1838 (sika deer) and *Capricornis crispus* (Temminck, 1845) (Japanese serows)	*Cervus nippon* (sika deer)	Horses	*Capricornis crispus* (Japanese serows)
**Female**									
Body width (at midbody)	200-225	NA	180-310	200-300	150-220	340	170-230	320-570	228-430
Distance between two adjacent ridges	150-250	NA	185-290	87-166	60	50-89	25-45	30*****	None
Shape of ridges (H/W)^†^ in longitudinal sections	13-18/18-25, Triangular	NA	8-23/23-30, Triangular	10-13/26, Rounded	3/15-23, Small, rounded	6/12****, Small	3/8****, Small, rounded	4/8*****, Small, rounded	None
Number of inner striae between adjacent ridges	None	NA	None	2-4	2	3-4****	2	2*****	None
Thickness of cuticle without ridges	25	NA	10-32	25-35*	10	20-30****	28-32	19*****	15-50******
Number of muscle cells per quadrant	8-13	NA	8-22	2-7**	5-7***	2-3****	1-2****	-	8-13******
**Male**									
Body width (at midbody)	NA	83-85x133-138	130x145	60-100	50-80	105	50-68	140-190	180-212
Thickness of cuticle	NA	5	4	5	2	-	-	-	8-10
Number of small ridges	NA	138-152	130	None	None	None	None	None	None
Small ridges (H/W)	NA	1/1	1/1	None	None	None	None	None	None

Dimensions in micrometres. ^†^H/W: height/width.

*-******: cited references.

**Figure 2:**
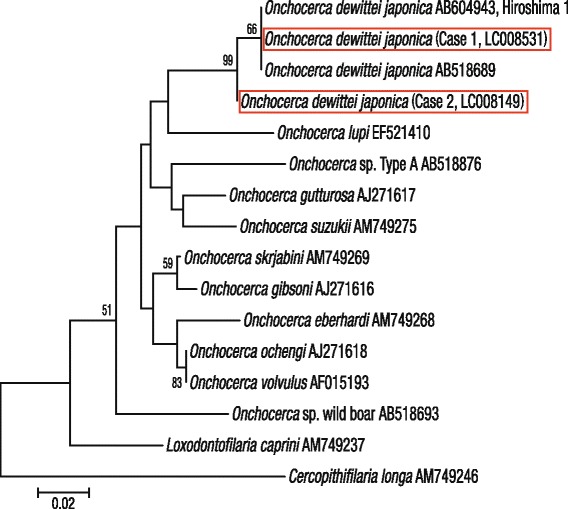
**Molecular identification of the causative agents (** ***Onchocerca dewittei japonica*** **) of Cases 1 and 2 (red rectangles).** Phylogeny of *Onchocerca* spp. based on GenBank sequences of the *cox*1 using the neighbour-joining method. The numbers at the nodes indicate the bootstrap confidence values on the basis of 500 replicates, where values >50% are shown. Bar, the number of changes inferred to have occurred along each branch.

The *cox*1 sequences (excluding primers) of our specimens were compared with those of 11 species that consisted of *Onchocerca* spp., *Onchocerca* sp. wild boar and *Onchocerca* sp. Type A from GenBank (Table 2). The nucleotide sequence of *cox*1 from the causative agent of Case 1 was identical to that of *O. dewittei japonica* from wild boars (Table 2 and Figure 2). Furthermore, in the phylogenetic tree, the causative agent of Case 1 and *O. dewittei japonica* were separated from other congeneric species, with a high bootstrap value (Figure 2). On the basis of the molecular analysis, we confirmed the causative agent of Case 1 to be *O. dewittei japonica*. In Case 2, the transverse sections of the midbody contained the seminal vesicle, intestine and the polymyarian muscle layer (Figure 1D). The seminal vesicle was filled with spermatozoids. We observed small longitudinal ridges (138–152 ridges) on the outer cuticle of the midbody, and the height/width of the ridges was 1/1 μm (arrows in Figure 1E). In the slightly oblique sections, small longitudinal ridges were observed (arrows in Figure 1F). The size and number of the ridges were identical or very similar to those of a male *O. dewittei japonica* (Table 1). Therefore, we identified the causative agent as a male *O. dewittei japonica*.

**Table 2:** **Differences in the nucleotide sequences (110 sites) of the** ***cox*** **1 gene among** ***Onchocerca*** **species**

		**1**	**2**	**3**	**4**	**5**	**6**	**7**	**8**	**9**	**10**	**11**	**12**	**13**	**14**
1	*Onchocerca dewittei japonica* (LC008531, Case 1 in the present study)		1	0	0	6	6	7	8	8	8	9	9	10	10
2	*Onchocerca dewittei japonica* (LC008149, Case 2 in the present study)	0.9		1	1	5	5	6	7	7	7	8	8	9	9
3	*Onchocerca dewittei japonica* AB604943, Hiroshima 1	0	0.9		0	6	6	7	8	8	8	9	9	10	10
4	*Onchocerca dewittei japonica* AB518689	0	0.9	0		6	6	7	8	8	8	9	9	10	10
5	*Onchocerca ochengi* AJ271618	5.5	4.5	5.5	5.5		0	3	3	4	8	3	5	8	8
6	*Onchocerca volvulus* AF015193	5.5	4.5	5.5	5.5	0		3	3	4	8	3	5	8	8
7	*Onchocerca skrjabini* AM749269	6.4	5.5	6.4	6.4	2.7	2.7		4	1	7	4	6	7	7
8	*Onchocerca eberhardi* AM749268	7.3	6.4	7.3	7.3	2.7	2.7	3.6		5	10	6	8	11	7
9	*Onchocerca gibsoni* AJ271616	7.3	6.4	7.3	7.3	3.6	3.6	9.1	4.5		7	5	6	8	6
10	*Onchocerca lupi* EF521410	7.3	6.4	7.3	7.3	7.3	7.3	6.4	9.1	6.4		7	7	10	13
11	*Onchocerca gutturosa* AJ271617	8.2	7.3	8.2	8.2	2.7	2.7	3.6	5.5	4.5	6.4		4	7	11
12	*Onchocerca suzukii* AM749275	8.2	7.3	8.2	8.2	4.5	4.5	5.5	7.3	5.5	6.4	3.6		7	10
13	*Onchocerca* sp. Type A AB518876*	9.1	8.2	9.1	9.1	7.3	7.3	6.4	10.0	7.3	9.1	6.4	6.4		11
14	*Onchocerca* sp. wild boar AB518693*	9.1	8.2	9.1	9.1	7.3	7.3	6.4	6.4	5.5	11.8	10.0	9.1	10.0	

Values above the diagonal are the numbers of nucleotide differences, and those below the diagonal are the percentages of nucleotide differences. *See text.

**Figure 3:**
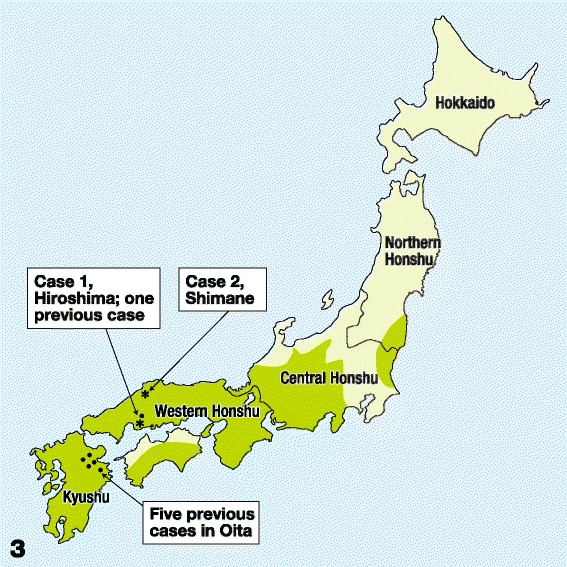
**Geographic distribution of human zoonotic onchocercosis in Japan.** Case 1 (*) in the current study and one previous case in Hiroshima; Case 2 (*) in Shimane; five previous cases reported in Oita, Kyushu. The green areas indicate habitat of wild boars in Japan in 2003 based on ‘The Sixth National Survey on the Natural Environmental Report of the Distributional Survey of Japanese Animals (Mammals)’ [25].

The nucleotide sequence of *cox*1 was compared with that of congeneric species from the GenBank (Table 2). With the exception of one of the 110 sequences, the nucleotide sequences of our specimen from Case 2 were identical to those of *O. dewittei japonica*, confirming that the causative agent was *O. dewittei japonica*. The areas where Cases 1 and 2 were found are indicated on the map of Honshu, Japan (asterisks in Figure 3).

## Discussion

We compared the morphological findings of causative agents obtained in the present study with those of congeneric species in Japan. For Case 1, the distance between the adjacent ridges and their triangular shape were very similar to that found in females of *O. dewittei japonica*, while differing from those of other species (Table 1). Furthermore, this specimen did not exhibit the inner striae in the middle layer of the cuticle that are typical for females of *O. gutturosa*, *O. lienalis*, *O. skrjabini*, *O. eberhardi* and *O. cervicalis* (Table 1) [5,21,32-36]. Females of *O. suzukii* lack the transverse ridges and inner striae in the cuticle [19]. We therefore diagnosed the causative agent of Case 1 as a female *O. dewittei japonica*.

To the best of our knowledge, most male *Onchocerca* spp. have no specific characteristics on their cuticle [21,35]. In contrast, males of *O. dewittei japonica* from Japanese wild boars and of *O. dewittei dewittei* Bain, Ramachandran, Petter & Mak, 1977 from Malaysian wild boars (*Sus scrofa jubatus* Miller, 1906) have small longitudinal ridges on the cuticle [20]. In Case 2, the transverse sections of the male worm had small ridges on the cuticle, and the number of ridges was similar to that found in a male *O. dewittei japonica* (Table 1). Among the 25 previously reported cases of zoonotic onchocercosis, only one case caused by a male worm has been reported (from Oita in Japan) owing to the worm’s morphological characteristics [16].

For Case 1, we verified that the histological sections stained with HE were available for molecular analysis using our primers. Our study indicated the first diagnosis of a male *O. dewittei japonica* confirmed by gene sequencing in Case 2. Moreover, the molecular analysis verified that the causative agents were different from unnamed *Onchocerca* spp. (*Onchocerca* sp. wild boar and *Onchocerca*. sp. Type A) found in Kyushu [22-24]. There was only one nucleotide difference between the causative agent of Case 2 and *O. dewittei japonica* obtained from wild boars in the current study (Table 2). According to Ferri et al. [37], the genetic distance between *cox*1 from two different species is greater than a threshold value of 4.8%. Therefore, we consider that this difference was intra-specific genetic divergence rather than inter-specific divergence. Furthermore, in the phylogenetic tree, the causative agents of Cases 1 and 2 and *O. dewittei japonica* had a high bootstrap value, which separated them from other congeneric species (Figure 2).

In the current study, Case 1 from Hiroshima and Case 2 from Shimane are the seventh and eighth reported cases of *O. dewittei japonica* infections in Japan, respectively (Figure 3). The causative agent of all six previous cases of zoonotic onchocercosis in Japan was *O. dewittei japonica* [5]. The five previous infections were concentrated in Oita, Kyushu, but another case was reported recently in Hiroshima, the western part of Honshu. Two infections in the current study and one previous case in Hiroshima indicated a new occurrence of the zoonotic onchocercosis in the western part of Honshu.

Regarding vectors of filarial parasites, Fukuda et al. [22-24] verified that the blackfly *S. bidentatum* both anthropophilic and zoophilic was a natural vector of *O. dewittei japonica* in Oita, Kyushu. First, they successfully infected blackflies with microfilariae through intrathoracic inoculations and detected third-stage larvae in blackflies. Second, from results of molecular studies, they identified the third-stage larvae of *O. dewittei japonica* in blackflies during field work in Oita. *Simulium bidentatum*, which is predominant in Kyushu, has been found in Honshu, Japan [4]. Therefore, it is speculated that the blackfly species also appears to be a vector of *O. dewittei japonica* in Honshu.

Fossil records and molecular analyses indicated that the ancestral population of wild boars in Southeast Asia expanded to East Asia and migrated to Kyushu Island and Honshu via southern bridges between the Korean Peninsula and Kyushu Island during the middle to late Pleistocene era (500,000–250,000 years ago) [38]. On the other hand, wild boars did not migrate to Hokkaido across northern bridges between Sakhalin and Hokkaido Island [39]. Therefore, wild boars did not naturally inhabit Hokkaido. In northern Honshu, wild boars were widely present during the Jomon period (12,000-2,400 years ago) but became extinct around 1900 owing to the climate change, actions by local government in response to heavy crop damage (1749 in Aomori Prefecture), expansion of rice fields and human habitat, infectious diseases (e.g. classical swine fever) and grey wolves (*Canis lupus* Linnaeus*,* 1758) as a natural enemy [40].

Recently, the habitat of wild boars extended 1.3 times its previous size in the western and central Honshu between 1978 and 2003, based on the national survey on the distribution of Japanese mammals (Figure 3) [25]. The number of wild boars in Japan has increased and is estimated to be several hundred thousand. The present expansion of wild boars was related to a decrease in the annual snowfall in Honshu since 1990 [41]. The annual snowfall in western Honshu is less than 70 days with a snow cover that is 30 cm deep, allowing the migration of wild boars for food. This trend of snowfall will continue in the near future owing to global warming [42]. In addition, unused rice fields have increased because the young workforce has migrated away from rural areas to urban areas. Such fields provide favourable areas for wallowing and feeding grounds for wild boars [43]. Likewise, the number of hunters has decreased because of advanced age [44]. Under these natural and social circumstances, wild boars increased their population and expanded their habitats. Wild boars, blackfly vectors and humans share the same habitat in rural areas in Honshu, as well as Kyushu.

Regarding the interaction between the prevalence of parasites in host animals and human infections, the high prevalence of *Thelazia* eyeworms in dogs should be considered as an indicator of infections in the human population [45,46]. Human infections of dirofilariosis have been reported in areas of high canine prevalence [47]. In northern Spain, the sero-prevalence rates on dirofilariosis in dogs and humans are almost identical: 12% in dogs and 11.6% in humans [48]. The infection rate of wild boars with *O. dewittei japonica* was found to be 78% in the Shimane Prefecture adjoining the Hiroshima Prefecture [5]. In Oita, Kyushu, five previous infections were found between 1989 and 2005. The prevalence of *O. dewittei japonica* in wild boars was 89% [5]. Therefore, the high prevalence of the causative agents in the host animals appears to be an important factor for zoonosis in humans. Consequently, further infections of zoonotic onchocercosis are likely to occur in the western and central parts of Honshu as well as Kyushu, Japan.

In this study, the presence of spermatozoids in the seminal vesicle indicates the maturation of the male *O. dewittei japonica* in humans. Likewise, a female *O. lupi* worm with microfilariae in the uterus was reported in a human case in Arizona [10]. This evidence suggests that these species of *Onchocerca* be able to adapt to humans. Chabaud and Bain [49] proposed that during the evolution of the genus *Onchocerca*, capture event (host-switching) was more important than their zoological affinity for host animals. According to Morales-Hojas [50], animal parasites may adapt to humans as a result of host-switching of parasites by vectors. We speculate that the causative agents of accidental zoonotic onchocercosis adapt to humans and evolve to cause new parasitic diseases in humans in the future.

## Conclusions

We identified causative agents in two cases of zoonotic onchocercosis in the western part of Honshu, the main Island of Japan. For Cases 1 and 2, we diagnosed the causative agents as a female and a male *O. dewittei japonica*, respectively, owing to their histopathological characteristics. The identity of the causative agents was confirmed by sequencing the *cox*1 gene. The present findings of zoonotic onchocercosis among the residents in Hiroshima and Shimane appear to reflect the high prevalence of *O. dewittei japonica* in wild boars in the western part of Honshu. Zoonotic onchocercosis is spreading throughout the western part of Honshu owing to the expansion of wild boar habitat in the area.

### Consent

Written informed consent was obtained from the patients for the publication of this report and any accompanying images.
